# Context-dependent immune regulation: a mechanistic and AI-enabled integrative framework

**DOI:** 10.3389/fimmu.2026.1850490

**Published:** 2026-07-10

**Authors:** Qingyu Chen, Yuxin Zhu, Yemeng He, Yaoyi Wang, Qianqi Xu, Ziyue Wang, Feifei Hu

**Affiliations:** 1 The Second School of Medicine, Wenzhou Medical University, Wenzhou, Zhejiang, China; 2 Renji College of Wenzhou Medical University, Wenzhou, Zhejiang, China; 3 School of Laboratory Medicine and Life Sciences, Wenzhou Medical University, Wenzhou, Zhejiang, China; 4 The First School of Medicine, School of Information and Engineering, Wenzhou Medical University, Wenzhou, Zhejiang, China; 5 School of Nursing and Midwifery, University of Galway, Galway, Ireland; 6 Department of Nephrology, The First Affiliated Hospital of Wenzhou Medical University, Wenzhou, Zhejiang, China

**Keywords:** artificial intelligence, context-dependent immune regulation, immune heterogeneity, multimodal integration, precision immunology, systems immunology

## Abstract

The immune system functions as a dynamic, multiscale, and context-sensitive network whose outputs depend on cellular composition, spatial organization, metabolic state, host background, and temporal trajectory. These properties have long been investigated through systems immunology, mathematical modeling, and immune simulation. This Perspective uses context dependence as an organizing principle to connect these foundations with recent artificial intelligence (AI) methods, and develops a context-centered view in which immune context is treated as an explicit, partially measurable, and partially uncertain state that can be encoded, constrained, and prospectively tested. It summarizes representative phenomena across cancer, infection, innate immunity, autoimmunity, and genetic variation; describes their microenvironmental, metabolic, systemic, and temporal determinants; and distinguishes the capabilities of mechanistic, data-driven, symbolic, and hybrid models. The proposed hybrid AI-mechanistic framework positions AI as a tool for context representation and multimodal integration, while mechanistic and knowledge-based components preserve explicit dynamics, biological constraints, and testable interventions. Practical strategies are discussed for limited biomaterial, partially longitudinal animal studies, missing or unpaired modalities, interpretability, and prospective validation. The framework does not require every modality in every sample; instead, it emphasizes minimum context annotation, uncertainty-aware integration, explicit assumptions, and benchmarking against simpler models.

## 1 Introduction

The immune system is a complex adaptive network whose functions span molecular signaling, cellular state transitions, tissue organization, and host-level physiology. Systems immunology has formalized this view by combining high-dimensional measurement with mathematical and computational representations of immune organization and dynamics (1, 2).

Nevertheless, immune effects are still frequently summarized as fixed properties of individual molecules, pathways, or cell types. Such summaries can obscure the extent to which an observed effect depends on cellular composition, spatial niche, metabolic resources, disease stage, treatment history, and host genetic background. The resulting contradictions are not always experimental noise; they may represent valid observations from different regions of a larger immune-state space (3–5). This problem is visible in heterogeneous responses to immunotherapy and in stimulation-specific genetic regulation of immune cells.

The central argument of this Perspective is that immune regulation should be understood as a conditional output of biological state rather than as a fixed intrinsic property of a molecule or pathway. In this framework, AI may be most useful when it complements mechanistic models by supporting context representation, multimodal integration, and hypothesis generation under explicitly stated assumptions. We therefore focus on how established modeling approaches and recent AI methods can be combined under realistic constraints, while recognizing that no single model class can represent every scale or compensate for unmeasured context.

To make this argument operational, we propose three linked propositions. First, immune effects should be modeled as conditional outputs of biological context rather than fixed properties of individual molecules, pathways, or cell types. Second, AI is most useful in this setting when it represents measured and missing context, instead of simply increasing model complexity. Third, hybrid AI-mechanistic models should be evaluated by whether they improve calibration, transportability, and experimentally testable hypotheses over simpler alternatives.

## 2 Existing foundations in systems immunology and mechanistic modeling

Mechanistic modeling provides explicit representations of how immune states change (6–9). Ordinary differential equation models describe continuous population or concentration dynamics, partial differential equations add spatial structure, stochastic models represent probabilistic events, and agent-based models simulate heterogeneous cells as interacting entities. Boolean and logical models describe regulatory state transitions when kinetic parameters are unavailable, whereas quantitative systems pharmacology and multiscale models connect drug exposure, cellular responses, tissue effects, and clinical outcomes.

These approaches make assumptions explicit, incorporate temporal behavior, and support counterfactual intervention. They have been applied to viral dynamics, signaling networks, cell-population interactions, tissue organization, and treatment response (10). They are not merely background references; they define the dynamic, mechanistic, and counterfactual questions that any AI-enabled framework should preserve.

Their limitations are also important. Parameter identifiability can be poor when measurements are sparse, model structures may not transfer across diseases or cohorts, and detailed multiscale models become difficult to calibrate as the number of components increases. High-dimensional imaging and multi-omics data exceed the practical input space of many manually specified models, while digital immune twins illustrate both the promise and the difficulty of linking individualized measurements to dynamic simulation (11, 12). These limitations motivate a complementary role for AI rather than the replacement of mechanistic modeling.

## 3 Context-dependent observations across immune settings

Context-dependent immune regulation means that signaling outputs, cell functions, and intervention effects are jointly determined by cellular state, spatial architecture, metabolic resources, host background, and temporal history. This principle reframes apparently conflicting observations as conditional effects that arise in different regions of an immune-state space rather than as evidence that every discrepancy has a single explanation (13).

Cancer immunity provides a familiar example. Tumors with similar immune-cell abundances may differ in spatial organization, cell-cell contacts, antigenicity, T-cell state, and myeloid architecture. Responses to checkpoint blockade therefore depend on a coordinated immune ecosystem and prior treatment context rather than on one universal marker (14–16). Recent perturbation-based therapeutic strategies further support this view. For example, ER-mitochondrial calcium shuttle-mediated calcipoptosis has been proposed as a way to induce immunogenic tumor-cell death and potent antitumor immunity, illustrating how intracellular perturbations may reshape immune responses in a context-dependent manner (17).

In infection and innate immunity, type I interferons can support early antiviral control but contribute to immunopathology or impaired antibacterial defense when their magnitude, cellular source, or duration is inappropriate. Trained immunity shows that prior microbial or inflammatory exposure can reprogram subsequent innate responses, whereas sepsis demonstrates that hyperinflammation and immune suppression may coexist or dominate at different stages. These settings require dynamic state-transition models rather than fixed pro-inflammatory or anti-inflammatory labels (18, 19).

Autoimmune and inflammatory diseases further demonstrate that pathway relevance depends on tissue, disease phase, and treatment background. Rheumatoid arthritis, inflammatory bowel disease, and systemic lupus erythematosus involve partially overlapping immune programs, yet their dominant mechanisms and therapeutic consequences vary across organs and patients (20–22). Host genetics is similarly conditional: many noncoding risk variants exert detectable effects only in particular cell types or activation states because chromatin accessibility and transcriptional circuitry change with exposure.

Immunological research should therefore move from asking only what a molecule or pathway does to asking under which conditions a specific effect is observed. This shift has direct implications for experimental design, data analysis, and clinical stratification: contextual variables should be recorded and modeled explicitly rather than treated as background noise. **Table 1** summarizes five representative domains and the modeling implications of their dominant contextual determinants.

**Table 1:** Representative context-dependent immune phenomena and their modeling implications.

Domain	Representative example	Dominant contextual determinants	Modeling implication
Cancer immunity	Heterogeneous response to checkpoint blockade	Tumor architecture, T-cell state, myeloid niche, prior treatment	Spatial and immune-state models should complement bulk biomarkers.
Innate immunity	Protective versus pathogenic type I interferon activity	Infection stage, cell type, signal magnitude, and duration	Time-dependent dynamic models are required to represent direction-changing effects.
Infection and sepsis	Hyperinflammation and immune suppression	Time, pathogen burden, organ dysfunction, and host baseline	State-transition models should replace single-stage disease labels.
Autoimmunity	Stage- and tissue-dependent inflammatory pathways in RA, SLE, and IBD	Disease phase, tissue compartment, treatment, and systemic inflammation	Stage-aware and tissue-aware models are needed for interpretation and intervention.
Host genetics	Context-specific eQTL and noncoding risk variants	Cell identity, activation state, stimulation, and chromatin accessibility	Genetic effects should be modeled as conditional rather than fixed covariates.

eQTL, expression quantitative trait locus; IBD, inflammatory bowel disease; RA, rheumatoid arthritis; SLE, systemic lupus erythematosus; TME, tumor microenvironment.

## 4 Mechanistic basis of context-dependence

Context-dependent immune regulation can be conceptualized as arising from three tightly coupled layers. The local microenvironment determines which signals immune cells receive, metabolic state determines whether and how those signals can be translated into functional outputs, and the host-level systemic state together with temporal trajectory sets the threshold, direction, and durability of the response. Together, these layers define the contextual state space in which immune regulation unfolds.

At the microenvironmental level, cellular composition, inflammatory status, spatial structure, and microbiota-derived signals are immediate determinants of immune outcomes. Spatial studies show that productive antitumor immunity depends not only on cell abundance but also on cellular neighborhoods and contacts. At barrier surfaces, type 17 responses may support homeostasis or chronic inflammation according to microbial composition, epithelial integrity, metabolite exposure, and host susceptibility (23).

At the metabolic level, nutrient availability and metabolic reprogramming determine whether immune cells can execute specific programs. Activated and quiescent immune populations differ in their use of glycolysis, fatty-acid oxidation, and oxidative phosphorylation, and these programs interact with signaling, epigenetic regulation, differentiation, and memory. Metabolism is therefore a component of immune state rather than a passive background variable (24, 25).

At the systemic and temporal levels, age, endocrine signals, prior infection, treatment exposure, organ-level interactions, and disease stage influence the threshold and durability of immune responsiveness (26). Time is not an external label: repeated exposure, recovery, chronic inflammation, and treatment pressure create path dependence. This helps explain why locally valid mechanisms may fail to generalize when host history and state transitions are omitted.

## 5 Computational approaches for context-dependent immunology

AI is not a single method, and different computational approaches represent different parts of immune complexity. Traditional machine-learning methods support feature selection, risk stratification, and heterogeneous treatment-effect estimation. Deep neural networks are flexible nonlinear function approximators, while transformer architectures can represent sequential dependencies when adequate longitudinal data are available (27–29). Recent single-cell foundation models and systems human immunology frameworks further extend this direction by learning cell-state or immune-state representations across large-scale multimodal datasets, although their mechanistic and causal validity still requires careful evaluation (30, 31). Flexibility alone does not guarantee biological validity, temporal fidelity, or reliable extrapolation.

Mechanistic models remain well suited to explicit hypothesis testing, dose-response relationships, temporal dynamics, and perturbation simulation when relevant variables and interactions can be defined. Data-driven models are more flexible for high-dimensional imaging, single-cell, spatial, and multimodal inputs, but they may learn cohort, platform, or sampling artifacts unless missingness, uncertainty, and validation design are handled explicitly (32).

Symbolic logic, semantic networks, knowledge graphs, and graph neural networks provide a relational layer. They can organize curated evidence or represent cells, genes, pathways, and tissue regions as connected entities, thereby linking learned patterns to candidate mechanisms. Their conclusions remain limited by the completeness and correctness of the underlying ontology, graph construction, and prior knowledge (33).

The most useful strategy is therefore question dependent and often hybrid. Mechanistic components contribute explicit dynamics, biological constraints, and intervention logic, while AI contributes scalable representation learning, parameter inference, surrogate modeling, and pattern discovery. Hybrid models can place learned components within mechanistic equations or constrain predictions with established network structure, but they still require transparent assumptions, out-of-distribution evaluation, uncertainty analysis, and experimental validation (34). The capabilities, strengths, limitations, and appropriate roles of the main computational approaches for context-dependent immunology are summarized in **Table 2**.

**Table 2:** Capabilities and limitations of computational approaches for context-dependent immunology.

Approach	What it captures	Strengths	Limitations	Role in this framework
ODE/PDE models	Continuous-time dynamics, feedback, population changes, and spatial gradients	Explicit and interpretable mechanism	Parameter dependent; difficult with high-dimensional heterogeneity	Model immune-response trajectories
Agent-based models	Individual-cell behavior and spatial interaction	Represents tissue microenvironments and cellular heterogeneity	Computationally intensive and difficult to calibrate	Simulate local immune ecosystems
Boolean/network models	Pathway states and regulatory logic	Useful when kinetic parameters are unavailable	Limited quantitative and temporal resolution	Represent signaling-state transitions
QSP/multiscale models	Drug, cell, tissue, and organism-level processes	Supports dose and intervention simulation	High construction and validation burden	Connect treatment exposure to multiscale response
Traditional machine learning	Associations between high-dimensional features and outcomes	Efficient prediction and feature prioritization	Often static and weakly mechanistic	Risk stratification and baseline comparison
Deep learning and transformers	Nonlinear, multimodal, and sequential structure	Flexible representation learning	Data intensive; vulnerable to opacity and shortcut learning	Infer immune-context representations
GNN/knowledge graph	Cell, gene, pathway, and spatial relationships	Natural representation of networks and neighborhoods	Depends on graph construction and knowledge quality	Link learned patterns to relational biology
Hybrid mechanistic-AI models	Data-driven patterns under biological constraints	Supports dynamic prediction and bounded counterfactual simulation	Requires careful calibration, validation, and uncertainty analysis	Integrate state inference with mechanistic interpretation

GNN, graph neural network; ODE, ordinary differential equation; PDE, partial differential equation; QSP, quantitative systems pharmacology.

## 6 Hybrid AI–mechanistic framework for immune-state inference

**Figure 1:**
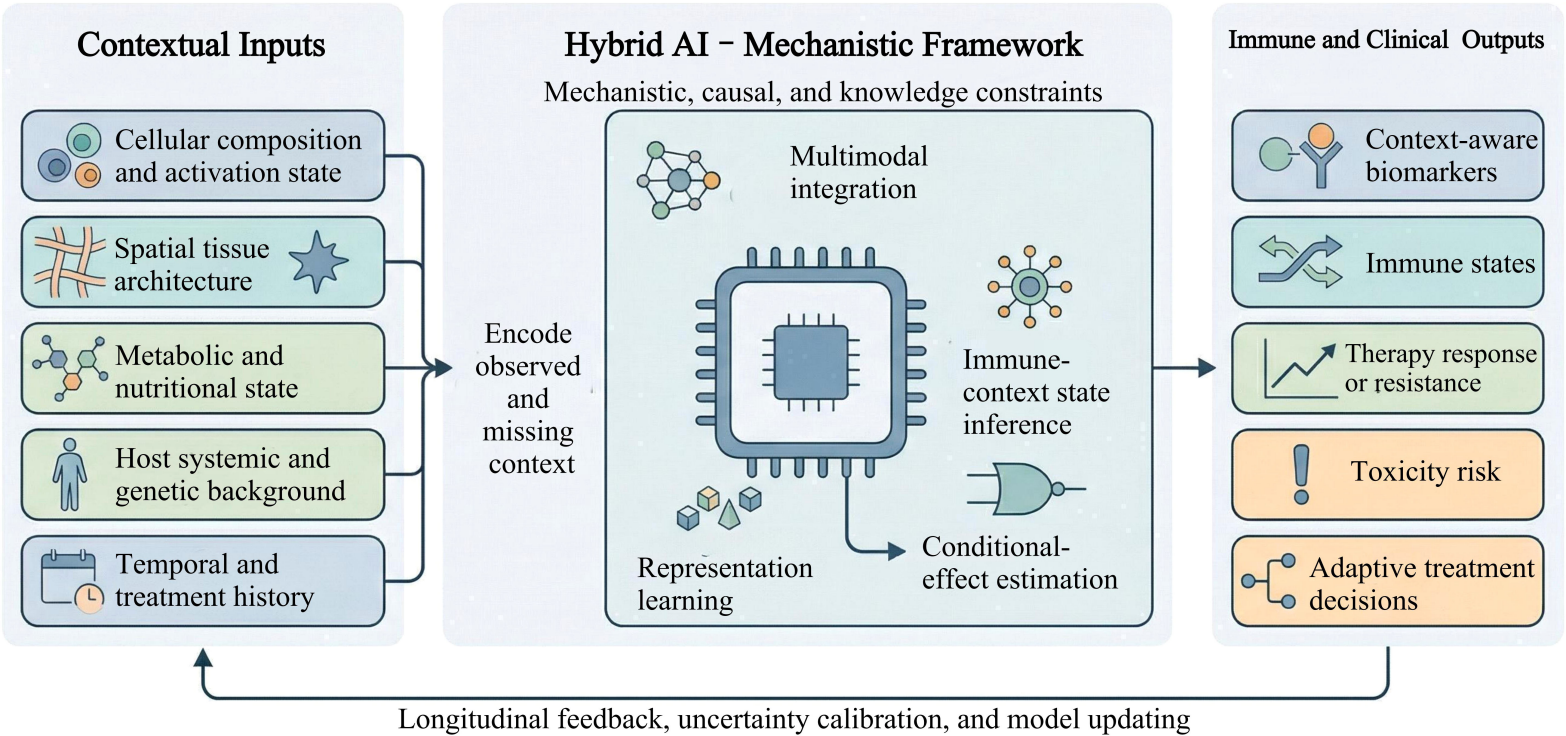
Hybrid AI–mechanistic framework for context-dependent immune-state inference. Immune outcomes are conditioned by cellular composition and activation state, spatial tissue architecture, metabolic and nutritional conditions, host systemic and genetic background, and temporal or treatment history. Available measurements are encoded together with explicit information about missing context. Data-driven models support representation learning and immune-state inference, whereas mechanistic models, causal structures, and knowledge graphs constrain state transitions and connect predictions to testable hypotheses. Longitudinal observations, when available, update the inferred state and support bounded simulation of alternative interventions. The framework emphasizes minimum context metadata, tiered measurement, uncertainty-aware integration, and disease-specific external and prospective validation.

The framework proposed here combines data-driven representation with mechanistic and knowledge-guided constraints. Its purpose is not to reconstruct a complete immune system from incomplete measurements or to replace established models, but to make observed and unobserved context explicit, infer task-relevant immune states, and connect predictions to bounded, testable interventions (**Figure 1**) (35).

The first step is context encoding. A minimum set of variables should be recorded even when comprehensive multi-omics is impossible: anatomical site, tissue or blood source, disease stage, treatment and infection history, age, sex, collection time, assay platform, and major pre-analytical factors. Additional layers may include cell-state composition, spatial neighborhoods, metabolic features, imaging, microbiome profiles, host genotype, and prior exposures.

The second step is multimodal integration and immune-state inference. Factor models, latent-variable approaches, nearest-neighbor alignment, and deep generative models can integrate transcriptomic, epigenomic, protein, spatial, and clinical measurements (36). The inferred state should be evaluated by its ability to generalize and support a prespecified task rather than by visualization alone. Recent benchmarking studies of single-cell multimodal integration methods further show that integration performance is highly task-, modality-, and metric-dependent, supporting the need for prespecified benchmarking rather than relying on integrated visualization alone (37). Missing modalities should be represented through partial observations and uncertainty rather than silently treated as measured.

The third step is mechanistic constraint and conditional-effect estimation. Equations, regulatory networks, causal graphs, and curated knowledge can restrict the set of plausible explanations for a learned state. Models may then estimate context-specific risks or heterogeneous treatment effects, but feature attribution alone should not be described as mechanism. Predictions should be translated into a limited set of perturbation hypotheses with stated conditions and failure criteria (27, 38). Emerging intracellular perturbation platforms, including biomolecular-condensate-based protein degradation tools, may provide experimentally tractable systems for testing model-derived intervention hypotheses (39).

The fourth step is longitudinal updating and bounded simulation. When repeated observations are available, immune states can be modeled as trajectories and used to update parameters or transition probabilities. Hybrid models may support counterfactual simulation, but outputs should distinguish what is constrained by observed data from what depends primarily on model assumptions (40).

## 7 Challenges and limitations

### 7.1 Limited biomaterial and tiered measurement

A human biopsy cannot routinely support comprehensive single-cell, spatial, proteomic, metabolomic, and functional assays. A tiered design is more feasible: minimum context metadata for all samples, scalable core assays for the full cohort, and deeper multimodal profiling in a deliberately selected subset. Peripheral blood, imaging, and circulating biomarkers may provide repeated or systemic information that complements, but does not replace, tissue measurements. Assay prioritization should be driven by the biological or clinical decision being modeled.

### 7.2 Partially longitudinal animal data

Terminal tissue collection prevents true within-animal longitudinal measurement for many organs. Parallel cohorts sampled at prespecified time points can characterize population-level dynamics, while repeated blood sampling, non-invasive imaging, and lineage tracing provide partial within-animal information. Trajectory reconstruction and pseudotime may organize cross-sectional observations, but they should not be described as equivalent to direct longitudinal follow-up.

### 7.3 Missing and unpaired multimodal data

Many available datasets contain measurements from different cells, samples, or cohorts. Integration methods can identify shared structure across partially paired modalities, but imputation may create overconfident biological narratives when a missing layer is weakly predictable. Missing-modality inference should therefore include held-out reconstruction tests, sensitivity analyses, uncertainty calibration, and comparison with models based only on directly observed variables (41). Benchmarking evidence reinforces that these tests should be task-, modality-, and metric-specific rather than assumed from global integration quality. Imputation should not be treated as measurement (42).

### 7.4 Interpretability and causal validation

Deep models may predict outcomes accurately while relying on non-causal correlates. Feature importance, attention weights, and saliency maps support inspection but do not establish mechanism. Stronger inference requires explicit causal assumptions, mechanism-informed constraints, negative controls, counterfactual analyses, and targeted perturbation experiments (43). Where these are unavailable, the model should be described as predictive and hypothesis-generating.

### 7.5 Prospective validation and clinical workflow

Retrospective discrimination is only an initial test of clinical value. Context-aware models should be evaluated across institutions, populations, assay platforms, and time periods, with prespecified handling of distribution shift and missingness. Prospective studies should assess calibration, decision consequences, workflow burden, fairness, and whether model use improves an actionable clinical process. Failure conditions should be reported alongside performance (44, 45). Clinical translation should also follow emerging consensus guidance for trustworthy and deployable healthcare AI, including transparent reporting, robustness assessment, usability, fairness, and post-deployment monitoring (46).

## 8 Future directions

A practical first step is to define minimum context annotation rather than an unattainable requirement for complete multi-omics. Shared reporting standards should distinguish measured, inferred, and unavailable context and should record sampling site, disease stage, treatment history, collection time, assay platform, and major pre-analytical factors.

Context-aware models should be evaluated against clear baselines. Relevant tests include whether contextual variables improve calibration or transportability over single biomarkers, whether longitudinal models outperform matched static models, and whether mechanistic constraints improve external validity or generate perturbation hypotheses that can be experimentally falsified. Additional variables should not be assumed to improve a model unless these gains are demonstrated.

Longer-term work may connect cross-organ interactions, immune memory, metabolism, and clinical outcomes in digital immune models. Such models should be developed in disease-specific and decision-specific stages, with explicit uncertainty and validation, rather than as unrestricted simulations of an entire patient. Their value will depend on whether they support reproducible inference and bounded intervention testing (11, 35).

## 9 Conclusion

Context dependence is an organizing principle of immune regulation: the effects of molecules, cellular pathways, and interventions are conditional on biological state, spatial organization, host background, and time. Systems immunology and mechanistic modeling have long provided tools for representing these dependencies. Recent AI methods add flexible approaches for learning high-dimensional representations, but their value depends on how well they preserve dynamic questions, expose assumptions, and connect predictions to biological tests.

Building on prior work in systems immunology, temporal modeling, and machine learning, this framework highlights several operational hypotheses for future benchmarking. Context-aware models should be compared with single biomarkers and disease-only baselines; longitudinal models should be compared with matched static models; and hybrid constraints should be evaluated by their effects on calibration, transportability, and experimentally testable predictions. Progress will depend less on measuring every possible modality than on using available data transparently, representing missing context and uncertainty, and validating models in the settings where they are intended to inform decisions.

## Data Availability

The original contributions presented in the study are included in the article/supplementary material. Further inquiries can be directed to the corresponding author.
